# Editorial note: repurposing FDA approved drugs as FXR agonists: a structure based in silico pharmacological study

**DOI:** 10.1042/BSR-2021-2791_EDN

**Published:** 2022-08-22

**Keywords:** agonists, FXR activators, molecular docking, molecular dynamics simulations

This Accepted Manuscript (published on 29 March 2022) is currently undergoing further peer-review before the final Version of Record is published.

After the acceptance of the manuscript, the authors requested to correct Figures 4, 5, 6 and 7. Given the significant corrections requested, the Accepted Manuscript and revised Figures are undergoing additional peer-review before the article's final publication. The Figures presented in the Accepted Manuscript may therefore be replaced in the Version of Record.

